# Anoxia tolerant DNA replication is supported by ATR kinase in the annual killifish *Austrofundulus limnaeus*

**DOI:** 10.1242/jcs.264892

**Published:** 2026-07-27

**Authors:** Riley Roth-Carter, Erin Helms, Joshua C. Saldivar, Jason Podrabsky

**Affiliations:** ^1^ Department of Biology, Portland State University, P.O. Box 751, Portland, OR 97207, USA; ^2^ Division of Oncological Sciences, Oregon Health and Science University, 2720 S Moody Ave., Portland, OR 97201, USA; ^3^ Department of Integrative Biology, Oregon State University, 2701 SW Campus Way, Corvallis, OR 97331, USA

**Keywords:** *Austrofundulus limnaeus*, Anoxia, ATR, Cell cycle, Replication stress

## Abstract

Hypoxia and anoxia suppress cell proliferation due to an increase in replication stress and activation of DNA damage checkpoints. Embryos of the annual killifish *Austrofundulus limnaeus* tolerate prolonged anoxia, indicating improved genomic stability under oxygen starvation. We investigated the cell cycle regulation of the anoxia tolerant killifish embryonic cell line PSU-AL-WS40NE during anoxia. Live-cell imaging confirms continued proliferation of WS40NE cells for the first 24 h of anoxia with minimal cell death. Fluorescence imaging shows that cells accumulate in G_1_ after the first day in anoxia with a rapid entry into S phase upon reoxygenation. Pharmacological inhibition shows a reliance on ataxia telangiectasia and Rad3 related (ATR) signaling, suggesting that increased γH2AX levels are driven by replication stress instead of DNA damage. This conclusion is supported by a lack of induction of a G_2_ checkpoint, suggesting minimal DNA damage during anoxic exposure. Maintaining cellular proliferation during anoxia and accumulating cells in the G_1_ phase for extended anoxic exposure is likely one way that embryos of the killifish can survive prolonged anoxia, which provides insight into mechanisms that enable cells to proliferate under metabolic stress.

## Teaser

**Summary:** Anoxia tolerant killifish cells continue DNA replication during extended anoxic exposure, accumulate in G1 during this exposure and rely on ATR to prevent replication stress and support recovery during reoxygenation.

## INTRODUCTION

Maintaining a stable genome is a vital requirement for cell and organismal survival and proliferation, with errors in processes involved in genome maintenance potentially leading to disease, dysfunction and death (Tomasetti et al., 2017; Zeman and Cimprich, 2014). Because of the potentially disastrous consequences of passing on genomic damage to daughter cells, there are numerous DNA damage and cell cycle checkpoints that function to facilitate genome repair prior to progression through the cell cycle (Boddy et al., 1998; Elledge, 1996). Cells are routinely able to withstand these basal levels of genotoxic stress but when the cellular environment changes, such as within hypoxic tumor microenvironments, genome maintenance pathways can become strained and unable to resolve the high levels of damage that can accumulate in these environments (Riffle et al., 2017). Detection of DNA damage is coordinated by the two core phosphatidylinositol 3-kinase-related kinases (PIKKs), ataxia telangiectasia mutated (ATM) and ataxia telangiectasia and Rad3 related (ATR), which when activated can lead to cells stopping at the G_1_/S checkpoint (Abraham, 2001). ATM protein is activated in response to double-strand breaks in the DNA, whereas ATR is activated in response to replication stress (Awasthi et al., 2015). Both proteins activate checkpoint kinases 1 and 2 (CHK1 and CHK2, also known as CHEK1 and CHEK2) and stabilize levels of p53 to induce cell cycle arrest through the up regulation of p21 (also known as CDKN1A) (El-Deiry et al., 1993). With DNA damage directly leading to exit from the cell cycle, it is expected that exposure to genotoxic stressors will cause a halt in the cell cycle until the stressor is removed and damage repaired. This strong control of the cell cycle prevents cell proliferation when DNA damage is present, and it also confirms replication does not continue when cells are exposed to stressful environments. Tolerance to high levels of environmental genotoxic stressors is a hallmark of metabolic dormancy in animals, as well as for radio-resistant human cancers and thus is an important area for continued research.

The effect of hypoxia and anoxia on control of the cell cycle is not completely understood. Hypoxia has been shown to activate p53 protein and leads to increased expression of p21 and p27 (CDKN1B) proteins, core proteins that inhibit cell proliferation, leading to cell cycle arrest (Druker et al., 2021; Hubbi and Semenza, 2015; Koshiji et al., 2004). At the same time, hypoxia has also been shown to cause an upregulation in aurora A (AURKA), a mitotic kinase, causing hypoxia-induced cellular proliferation (Klein et al., 2008). Cell proliferation during exposure to stressful conditions, especially hypoxia and anoxia, is known to lead to high mutational burden and in cancers is a driver of malignant progression (Vaupel, 2004).

Embryos of the annual killifish are extremely tolerant to many different known genotoxic stressors, such as high dose UV-C and ionizing radiation, 3% H_2_O_2_ and months of complete anoxia (Podrabsky et al., 2016, 2007; Wagner et al., 2019; Wagner and Podrabsky, 2015). These conditions are known to cause DNA damage and should induce withdrawal from the cell cycle based on data from mammalian systems. Primary cells that have been isolated from embryos of the annual killifish in normal conditions appear to be mostly in the G_1_ phase of the cell cycle and presumably quickly pass through S and G_2_. This is in stark contrast to embryos from other fish species where many cells are found in S and G_2_ (Meller et al., 2012). When the embryos are exposed to 24 h of anoxia there is no effect on the percentage of cells within G_1_ and G_2_ when compared to normoxic conditions (Meller et al., 2012). This accumulation of cells in G_1_ is hypothesized to allow for decreased energy cost for genome maintenance during prolonged anoxic exposure. This signature could be caused by cells immediately arresting at the stage they are in when exposed to anoxia, as seen in other stress-tolerant organisms, such as the embryos of the soil nematode *Caenorhabditis elegans*, which also enter into a dormant state and exit from the cell cycle when exposed to anoxic conditions (Hajeri et al., 2005). Embryos of the zebrafish *Danio rerio* can also arrest when exposed to anoxia, but seem to only arrest in the S and G_2_ phases of the cycle (Padilla and Roth, 2001). The high proportion of G_1_ phase cells in *A. limnaeus* embryos suggests a potentially unique regulation of the cell cycle during development and in response to stress. However, previous studies have relied on DNA content alone to define cell cycle kinetics, limiting the ability to draw more mechanistic conclusions concerning cell cycle regulation in response to anoxia.

To better study cellular mechanisms associated with this anoxia tolerance, in this study, we use an immortal cell line isolated from late-stage *A. limnaeus* embryos denoted PSU-AL-WS40NE (Riggs et al., 2019). This cell line exhibits a strong tolerance of anoxia, with many cells surviving 7 weeks without oxygen at 30°C (Riggs et al., 2019). Along with this impressive anoxia tolerance these cells appear to continue to proliferate for several days when exposed to anoxia based on cell counts (Riggs et al., 2019). Continuing to replicate DNA and proliferate while exposed to these genotoxic stressors would presumably cause large levels of DNA damage and cell death, as is seen in other systems (Vaupel, 2004). Yet, WS40NE cells exposed to anoxia and reoxygenation can resume normal cellular functions and proliferation. Discerning the ability of the annual killifish to continue cell proliferation during anoxic exposures without deleterious effects, shown by continued normal embryonic development after extended anoxia and reoxygenation, could lead to a better understanding of the role of cell cycle regulation in the extreme anoxia tolerance of these cells and might help determine the drivers of radio- and chemo-resistance in cancers that exhibit similar cell stress responses to oxygen deprivation (Podrabsky et al., 2007).

## RESULTS

### PSU-AL-WS40NE cells continue cellular replication during anoxia and reoxygenation

**Fig. 1.**
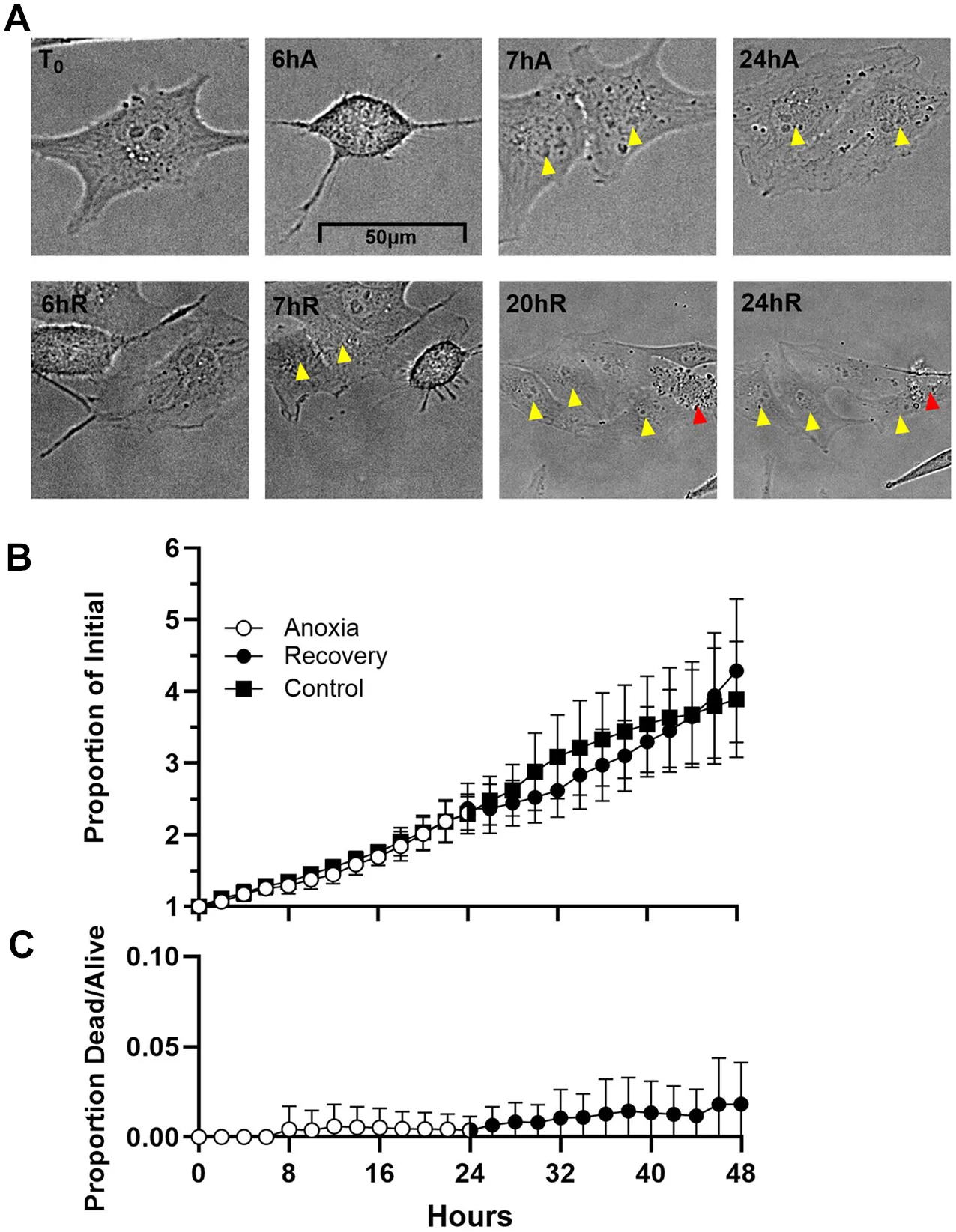
**Live bright-field imaging of WS40NE cells during anoxia and reoxygenation.** (A) A single cell folowed through both anoxia (A label, 0–24 h) and reoxygenation (R label, 30–48 h). Yellow arrowheads indicate surviving daughter cells and red arrowheads indicate morphologically dead daughter cells. (B) Quantification of cell counts during anoxia and reoxygenation and a normoxic control as a proportion of the initial cells. Results presented as mean±s.d., *n*=3. (C) Quantification of the proportion of morphologically dead cells compared to live cells at that same timepoint. Results presented as mean±s.d., *n*=3.

To confirm and expand on previous data suggesting that the cell line PSU-AL-WS40NE (hereafter WS40NE) is able to continue proliferation during initial anoxic exposure, live-cell bright field images were collected over a time-course of 24 h of anoxia followed by 24 h of reoxygenation to visualize cellular morphology, behavior and divisions. These images showed clear cell divisions occurring during anoxia and survival of the daughter cells throughout the anoxic exposure (Fig. 1A). Daughter cells produced during anoxia were also able to divide again following reoxygenation (Fig. 1A). Cell counts of these images revealed that cellular proliferation continued throughout the entire 24 h of anoxia at similar rates to those seen for cells in normoxia, suggesting that there is no pause in the cell cycle within the first 24 h of anoxia. This trend continued through reoxygenation as well, although there might be a slight depression in early time points of reoxygenation followed by a slight increase in overall proliferation after 24 h of reoxygenation compared to what is seen with 48 h of normoxia (Fig. 1B). Based on cellular morphology, less than 2% of cells experienced cell death during the time-course of anoxia and aerobic recovery (Fig. 1C).

### Anoxia induces delayed G_1_ arrest with rapid and sustained S phase increase after reoxygenation

**Fig. 2.**
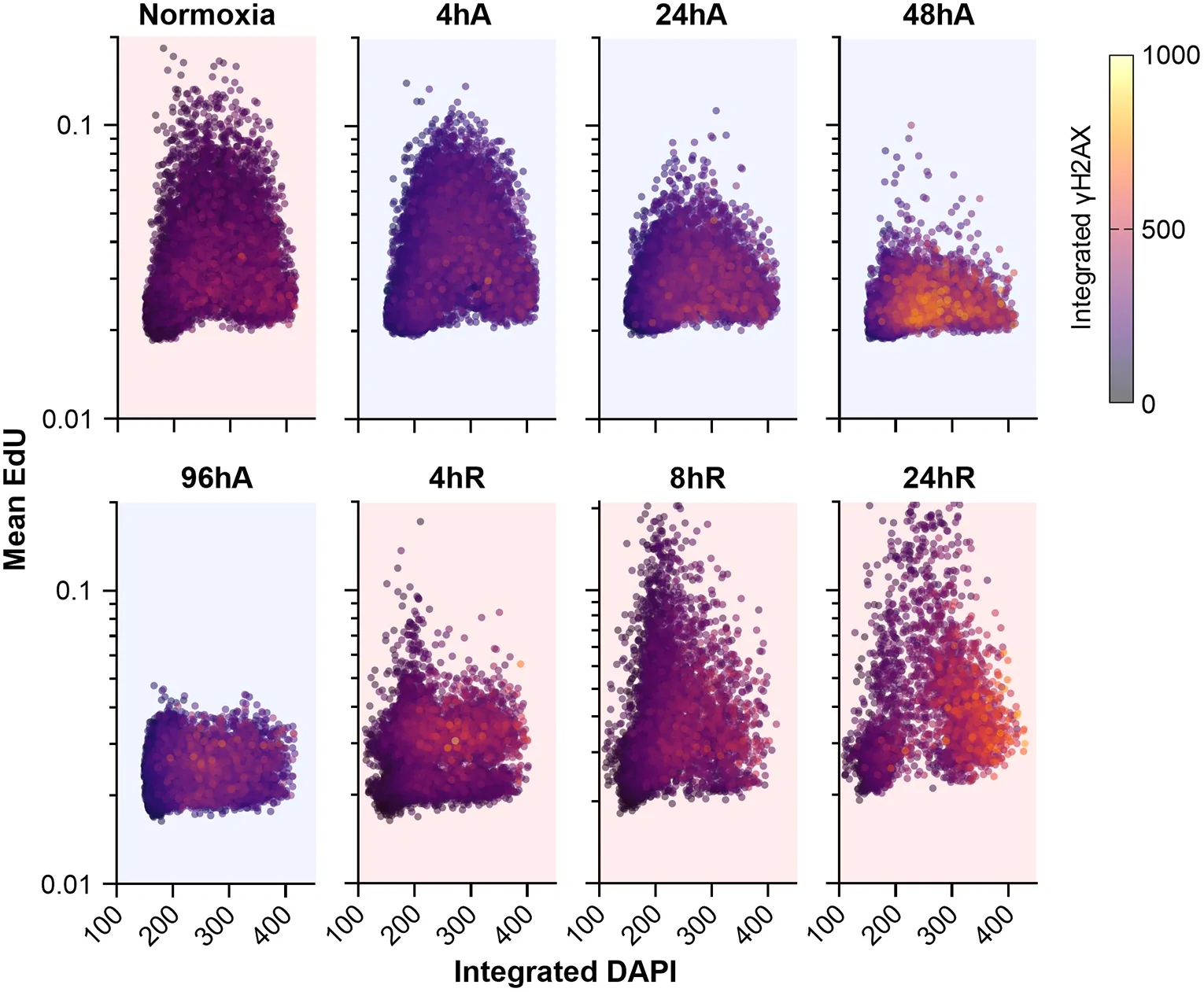
**Quantitative image-based cytometry data of WS40NE cells during anoxia and reoxygenation.** WS40NE cells were plated at 1.5×10^5^ cells per well in a glass-bottom optical 96-well plate and then exposed to anoxic conditions for 96 h followed by 24 h of normoxic conditions. Each dot represents the EdU and DAPI fluorescence for an individual cell, with dot color indicating levels of γH2AX. Red background indicates normoxic control and reoxygenation, blue background indicates anoxic conditions. Graph axes use arbitrary fluorescence units.

**Fig. 3.**
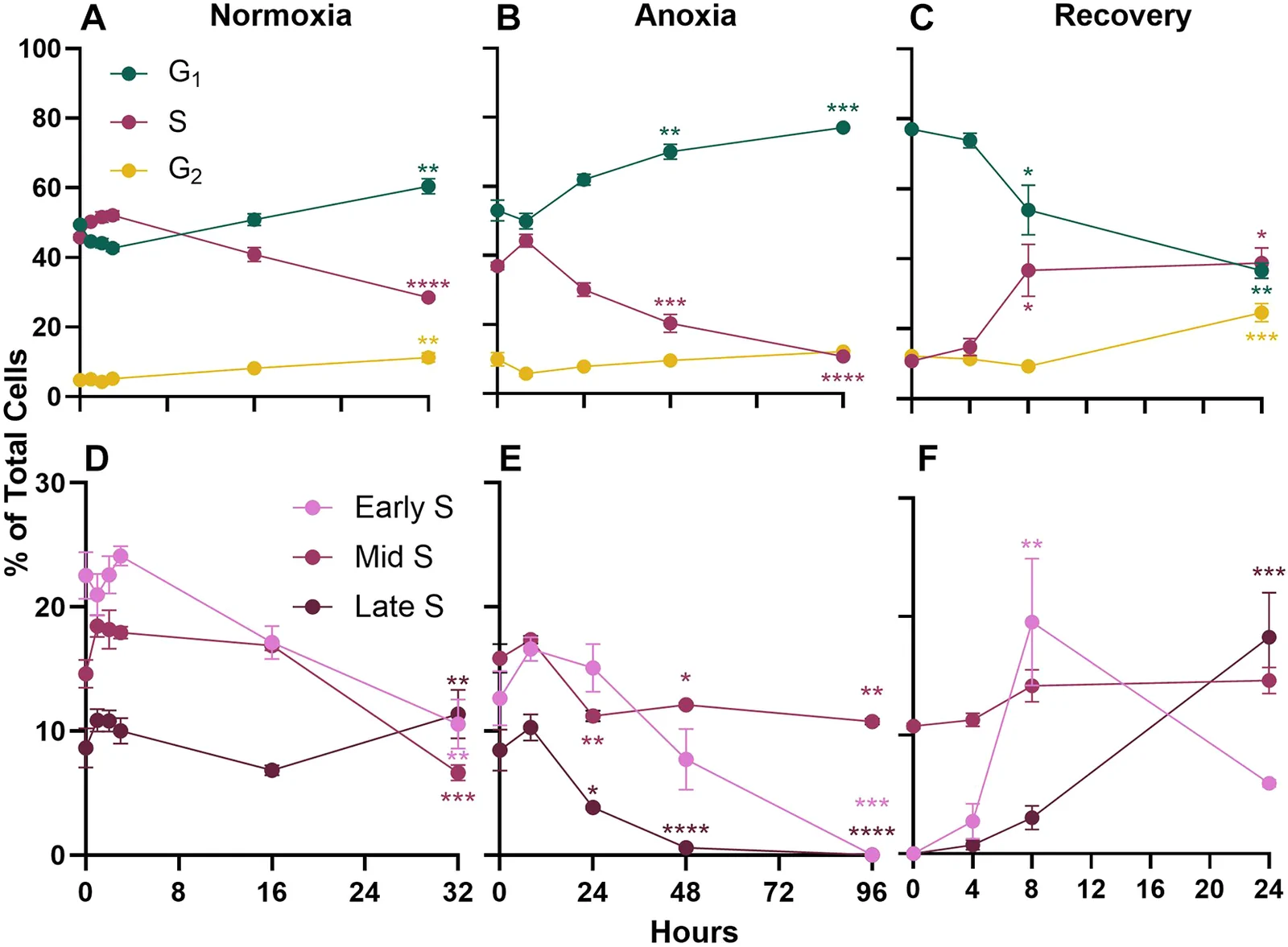
**Cell cycle analysis of WS40NE cells exposed to anoxia and reoxygenation.** Proportion of cells within each stage of the cell cycle were quantified during anoxia and reoxygenation. Proportion of each stage of the cell cycle in cells exposed to: (A) normoxia for 32 h or (B) anoxia for 96 h followed by (C) reoxygenation for 24 h. (D–F) The proportion of cells in early-, mid-, and late-S phases during (D) normoxia, (E) anoxia and (F) reoxygenation. Results presented as mean±s.e.m., *n*=3. *P<0.05; **P<0.01; ***P<0.001; ****P<0.0001 (one-way ANOVA with Dunnett's post hoc test comparing all time points within a single stage to the 0 h point within that condition).

With evidence of continued cell proliferation during short exposures to anoxic conditions, we wanted to determine the effect of longer anoxic exposure and get improved resolution into the cell cycle during anoxia. To do this we utilized quantitative image-based cytometry (QIBC) with EdU pulsing to quantify cell cycle dynamics across a time course of anoxia and reoxygenation in WS40NE cells (Luis et al., 2013). We also monitored levels of phosphorylation of the histone variant H2AX (γH2AX), a marker for DNA damage and replication stress. EdU incorporation is significantly reduced after 24 h of anoxia and by 96 h almost all EdU incorporation has stopped (Fig. 2). Interestingly, we found that after only 4 h of reoxygenation there was already a population of cells restarting S phase, as indicated by EdU incorporation, and by 24 h we saw a return to normal levels of EdU incorporation in S phase cells. Analysis of cell cycle phases revealed normoxic cells to have relatively equal levels of G_1_ and S phase cells, with 49.6% and 45.7%, respectively, and only a small proportion (4.7%) of cells in G_2_ (Fig. 3A). As cells grew towards confluence in normoxia, there was a slow trend towards an increase in G_1_ phase and decrease in S phase cells until there was a majority (60.4%) of cells in the G_1_ phase of the cell cycle with 28.6% and 11% of cells in S and G_2_, respectively, after 32 h (Fig. 3A). This end timepoint of normoxia is in line with previous analysis of whole embryos of the annual killifish, which found ∼80% of cells in G_1_ (Meller et al., 2012). Interestingly, after initial exposure to anoxia there was a stabilization or perhaps even a slight increase in the percentage of S phase cells after 4 h, suggesting sustained and perhaps even increased DNA replication during the initial transition into anoxia (Fig. 3B). The number of cells actively replicating their DNA (in S phase) then slowly declined, reaching a low of ∼20% after 48 h and 12% after 96 h of anoxia (one-way ANOVA with Dunnet's post hoc test, *P*=0.0009 and *P*<0.0001, respectively). As S phase cells decreased, there was an increase in G_1_ phase cells to ∼70% of cells after the first 48 h and 77% by 96 h of anoxia (one-way ANOVA with Dunnet's post hoc test, *P*=0.0025 and *P*=0.0004, respectively). There was little change in the level of cells in G_2_ at 96 h of anoxia compared to normoxic levels, comprising the remaining 12% of cells (one-way ANOVA with Dunnet's post hoc test, *P*=0.001).

After 4 days of anoxia, we reintroduced cells to normoxic conditions to observe the effect of reoxygenation on cell cycle dynamics. These data, coupled with the low number of dead cells reported in Fig. 1, support a quick re-entry of cells into the cell cycle and resumption of DNA replication after 8 h of aerobic recovery (Fig. 3C; one-way ANOVA with Dunnet's post hoc test, *P*=0.003). This increase was accompanied by a comparable decrease in the G_1_ population (*P*=0.03) at 8 h. This large increase in S phase cells and comparable decrease in G_1_ was maintained for the first 24 h of recovery. Interestingly, there was also a significant increase in G_2_ cells after 24 h of recovery (*P*=0.003). If S phase cells were divided into early, mid and late based on DNA content (Fig. 3D–F), a marked increase in early S phase cells was observed after 8 h of aerobic recovery (Fig. 3F; *P*=0.003). These data are consistent with cells entering S phase after arrest at the G_1_/S checkpoint during anoxic exposure.

### Extended anoxic exposure causes increased γH2AX

**Fig. 4.**
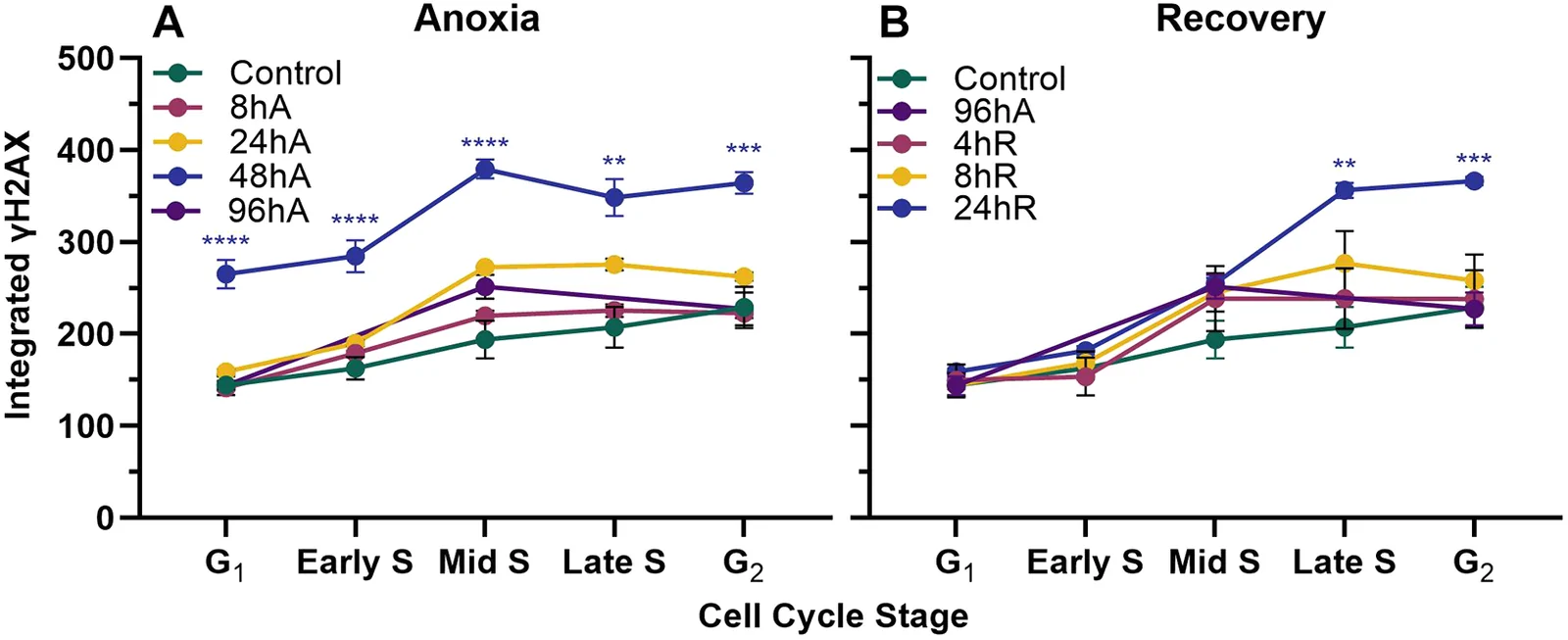
**Quantification of γH2AX during anoxia and reoxygenation across cell cycle stages.** Integrated γH2AX signal from QIBC images taken across (A) 96 h of anoxia and (B) 24 h of reoxygenation. Results presented as mean±s.e.m., *n*=3. **P<0.01; ***P<0.001; ****P<0.0001 (one-way ANOVA with Dunnett's post hoc test comparing all time points within a single cell cycle stage to control within that condition).

This continuation of proliferative cell cycle dynamics for the first 48 h of anoxia is intriguing in the context of increased levels of γH2AX, a marker of both replication stress and DNA damage (Figs 2 and 4A). At 48 h of anoxia there was a significant increase in γH2AX in all stages of the cell cycle. Interestingly by 96 h of anoxia this increase in γH2AX was completely lost. During recovery, the only significant increase in γH2AX was seen at 24 h of recovery and only in late-S and G_2_ cells (Fig. 4B).

### Increased γH2AX is driven by replication stress

**Fig. 5.**
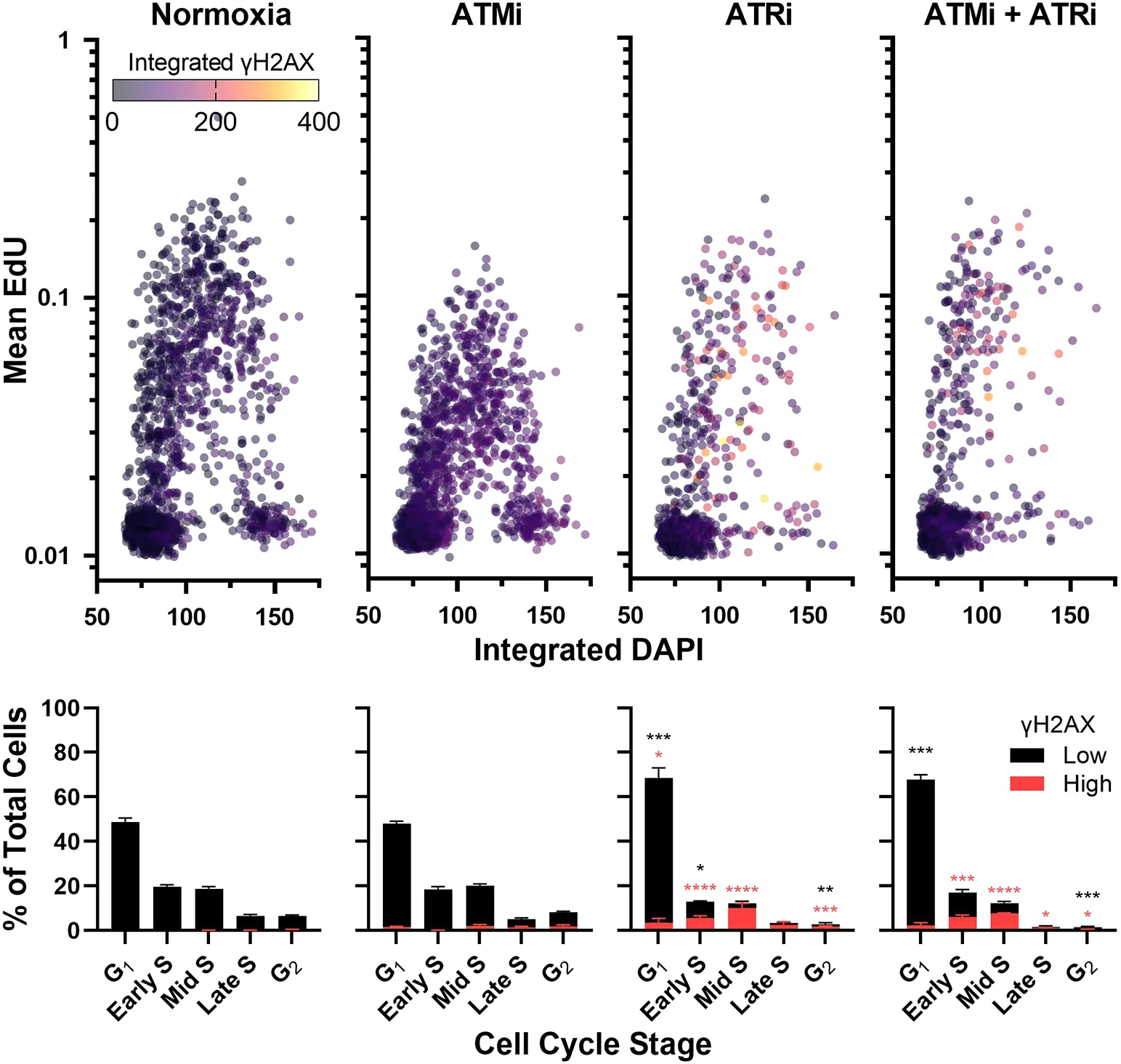
**Effect of phosphatidylinositol 3-kinase-related kinase inhibitors on the cell cycle and γH2AX after 24 h of normoxia.** WS40NE cells were exposed to inhibitors of ATR (8 μM AZ20) and ATM (0.01 μM AZD0156) in normoxic conditions for 24 h. Top panels, each dot represents EdU and DAPI fluorescence for an individual cell, with dot color indicating levels of γH2AX. Graph axes use arbitrary fluorescence units. Bottom panels, each cell cycle stage quantified as proportion of total cells and the proportion of high γH2AX cells within each stage. Cells are indicated as γH2AX high if integrated fluorescence intensity is higher than normoxic controls. Results presented as mean±s.d., *n*=3. *P<0.05; **P<0.01; ***P<0.001; ****P<0.0001 (one-way ANOVA and Dunnett's post hoc test comparing proportions of each cell stage and high versus low γH2AX to control; black asterisks represent changes in cell cycle stage proportion and red asterisks represent changes in proportion of high versus low γH2AX).

**Fig. 6.**
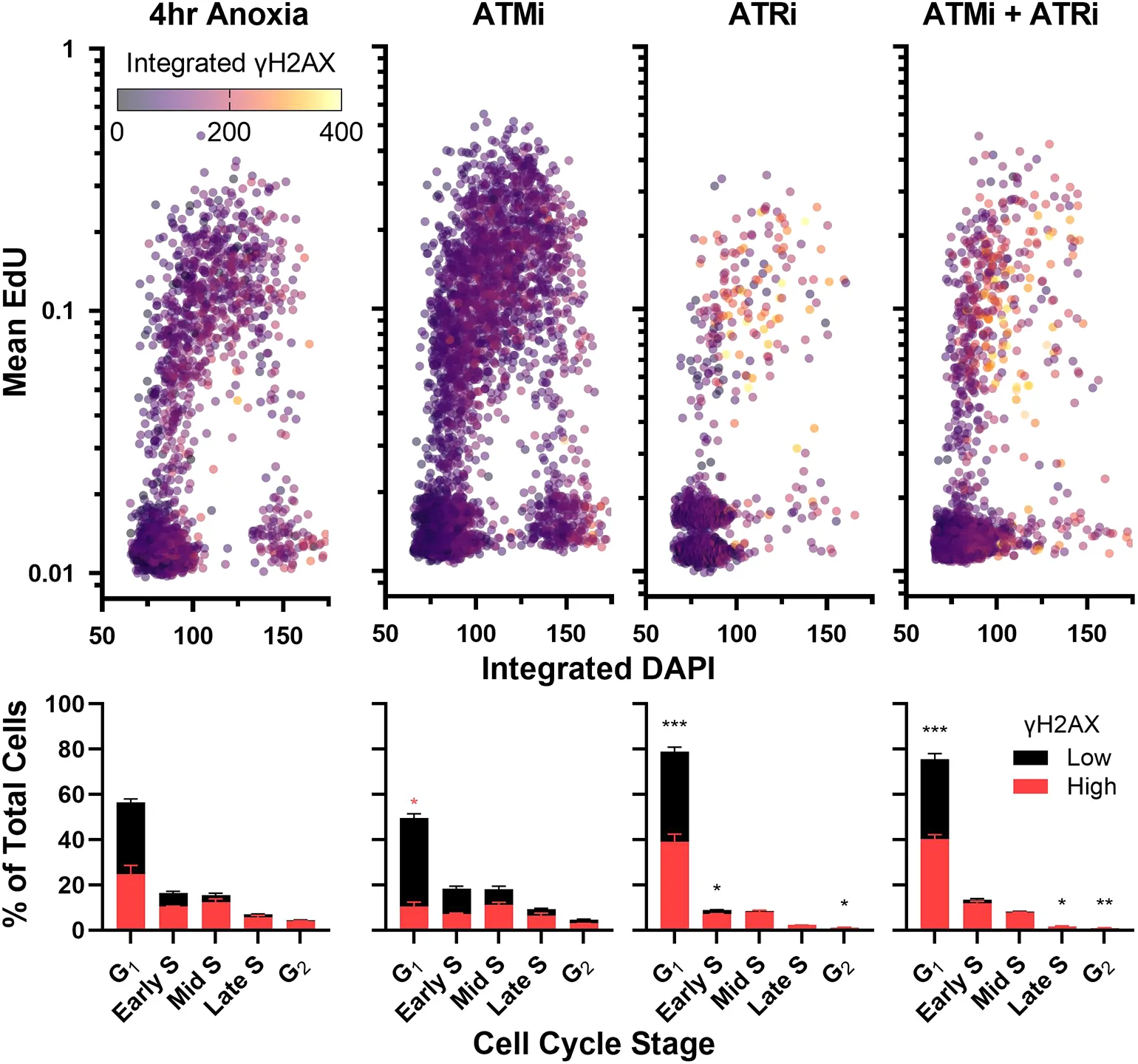
**Inhibition of PIKKs affects cell cycle kinetics at 4 h of anoxia.** WS40NE cells were exposed to inhibitors of ATR (8 μM AZ20) and ATM (0.01 μM AZD0156) for 1 h of normoxia and then 4 h of anoxia. Top panels, each dot represents EdU and DAPI fluorescence for an individual cell, with dot color indicating levels of γH2AX. Graph axes use arbitrary fluorescence units. Bottom panels, each cell cycle stage quantified as proportion of total cells and the proportion of high γH2AX cells within each stage. Cells are indicated as γH2AX high if integrated fluorescence intensity is higher than normoxic control. Results presented as mean±s.d., *n*=3, *P<0.05; **P<0.01; ***P<0.001 (one-way ANOVA and Dunnett's post hoc test comparing proportions of each cell stage and high versus low γH2AX to control; black asterisks represent changes in cell cycle stage proportion and red asterisks represent changes in proportion of high versus low γH2AX).

As the increase of γH2AX seen during anoxia can either be attributed to an increase in replication stress or DNA damage, we used small-molecule inhibitors of the key kinases ATR and ATM to test for the source of γH2AX. During anoxia and normoxia, ATM had no clear effect on either the distribution of cells across the cell cycle or the intensity of γH2AX (Figs 5 and 6). By contrast, the inhibition of ATR caused an increase in G_1_ phase cells during normoxia, and an increase in γH2AX intensity in G_1_ (Fig. 5). These increases are likely due to the mitotic transmission of under-replicated DNA to the daughter G_1_ cells (Chanoux et al., 2009; Guo et al., 2000; Pedersen et al., 2015). Strikingly, ATR inhibition under anoxia causes a dramatic increase in γH2AX in S phase cells (Fig. 6), suggesting anoxia tolerance is dependent on ATR signaling to prevent replication fork collapse and nucleus-wide DNA damage. Taken together, this suggests that anoxia does not cause significant amounts of DNA damage in S phase, but rather increases replication stress, which is addressed by ATR signaling to prevent global replication fork collapse.

## DISCUSSION

Here, we report that the WS40NE cell line transitions into anoxia without major disruptions to cell cycle progression or DNA replication. Rather, they react through a slow and controlled process that results in cells accumulating in G_1_. This is a unique pattern when compared to other vertebrate systems. Hypoxia and anoxia typically induce a rapid arrest in cell proliferation, likely an adaptive response to prevent increased cell populations and thus increased oxygen demand within tissues, with continued hypoxic exposure leading to cell death (Baldea et al., 2018; Carmeliet et al., 1998; Gardner et al., 2001; Goda et al., 2003; Hubbi and Semenza, 2015). However, this general response is not universal, with many cancers and stem cell populations showing increased cell proliferation during hypoxic exposure (Gordan et al., 2007; Hubbi and Semenza, 2015; Morrison et al., 2000).

Continued cellular proliferation with slow loss in S phase cells during anoxic exposure in WS40NE cells is similar to what is observed in the gills of the anoxia-tolerant Crucian carp, *Carassius carassius* (Sollid et al., 2005), although in the Crucian carp there is not a rapid increase in S phase cells after reoxygenation. However, it is worth noting that, at the organismal level, killifish embryos and Crucian carp have differing strategies in response to anoxia. Carp remain active during anoxia and rely heavily on temperature-driven metabolic depression and glycolytic energy production, while killifish embryos enter a profound state of metabolic and developmental dormancy. Thus, the continued cell proliferation in killifish cells might be viewed as an adaptive mechanism to support reversible cell cycle arrest, rather than a continued proliferation supported by changes in metabolic pathways.

In most vertebrate cells, mismatch between ATP production and consumption during anoxia leads to an energetic crisis and induction of cell death (Taylor and Pouyssegur, 2007). In contrast, during reoxygenation an increase in cellular reactive oxygen species (ROS) is the driver of cell death (Eleftheriadis et al., 2021; Neri et al., 2017). In WS40NE cells, exposure to anoxia and reoxygenation do not cause high levels of cell death, especially when compared to other vertebrate models (Azad et al., 2008; Carmeliet et al., 1998; Lefevre et al., 2017; Zhu et al., 2010). Thus, WS40NE cells show a remarkable capability to survive without oxygen and to maintain viability through reoxygenation. There is evidence that cancer cells respond to hypoxic exposure by preparing for reoxygenation, which then assists in rapid recovery and cellular proliferation after they return to normoxic conditions (Schlaepfer et al., 2015). It is likely that the WS40NE cells are responding in a similar way, as evidenced by the rapid increase of S phase cells beyond that observed in normoxia as an early response to reoxygenation. In whole embryos this rapid proliferation after reoxygenation could be vital to repair damaged tissues or could be a compensatory response to increase developmental rate post-anoxia.

The ability to maintain cellular proliferation during hypoxic and anoxic exposures requires functioning DNA damage repair pathways. *H2afx^−/−^* mice, which have a deletion of the gene encoding for histone H2AX and thus cannot produce γH2AX, lose hypoxia-driven endothelial cell proliferation (Economopoulou et al., 2009). In most cells, hypoxia is a known driver of replication stress, and the DNA repair response is required to efficiently restart stalled replication forks (Ng et al., 2018). Consistent with this, our data show that the replication stress response kinase ATR protects S phase cells from accumulating DNA damage allowing DNA replication for several hours in anoxic conditions. However, WS40NE cells eventually reduce replication. Extended anoxia causes a G_1_ arrest and a return of γH2AX levels to near normoxic levels after 4 days of anoxia. This suggests the completed repair of DNA damage and the accumulation of cells that are prepared to proliferate almost immediately upon reoxygenation. Notably, even with both ATR and ATM inhibition during anoxia exposure, high levels of γH2AX are observed. This suggests activation of the third protein in the family of kinases that can phosphorylate H2AX, DNA-PKcs (An et al., 2010).

In conclusion, WS40NE cells are able to maintain normal cellular proliferation for up to 48 h of anoxic exposure, even with apparent increased replication stress. This anoxia tolerance is associated with sustained DNA replication for several hours and a reliance on the replication stress response kinase ATR to prevent widespread DNA damage in S phase. Continued proliferation potentially allows the cells to reach a stage of the cell cycle that promotes survival of extended anoxic exposures and prepares them for recovery during reoxygenation. Further work is needed to confirm if this response is also observed in the whole embryo, as cells in culture might rely on a different strategy to survive anoxic stress compared to whole embryos. Anoxia and reoxygenation are an integral part of several different pathological states and environmental conditions, and better understanding the molecular mechanisms that support WS40NE cells to survive these stressors holds promise for the development of new interventions and therapies.

## MATERIALS AND METHODS

### PSU-AL-WS40NE cell culture

The PSU-AL-WS40NE cell line is a neuroepithelial line derived from Wourms' stage 40 embryos of *Austrofundulus limnaeus* (Riggs et al., 2019). Cells were maintained in complete cell culture medium consisting of Gibco™ Leibovits's L-15+Glutamine (11-415-114, Thermo Fisher Scientific, Waltham, MA, USA), 8.5% fetal bovine serum (FBS; 97068-085, VWR, Radnor, PA, USA), 5 mM glucose (45001-116, VWR, Radnor, PA, USA) and 100 U/ml penicillin/streptomycin (pen/strep; 15-140-122, Thermo Fisher Scientific) in 100×20 mm CytoOne™ Tissue Culture dishes (CC7682-3394, USA Scientific, Ocala, FL, USA) at 30°C under normal atmospheric conditions. For experiments, cells were grown in 96-well optical culture plates designed to be used for imaging (P96-1.5P, Cellvis, Mountain View, CA, USA).

### Anoxic exposure

Anoxic cell culture medium was prepared as previously described (Riggs et al., 2019). Briefly, pen/strep and glucose were added to L-15 to a final concentration of 100 U/ml and 5 mM, respectively. The required amount of FBS to reach a final concentration of 8.5% was injected into a sterile hydrated Slide-A-Lyzer™ dialysis cassette (A52964, Thermo Fisher Scientific) using a sterile syringe and 22 gauge needle. The cassette was then placed within the L-15 medium and bubbled for 1 h with sterile-filtered (0.22 μm) high purity nitrogen gas before being transferred into an anaerobic chamber (Bactron EZ, Sheldon Laboratories, Cornelius, OR, USA) with an atmosphere of 5% H_2_ and 95% N_2_. The medium was bubbled within the chamber overnight with 0.22 μm filtered chamber gas mixture using an aquarium pump. The following day, the FBS was withdrawn from the cassette with a sterile needle and syringe and added to the L-15 medium. The complete medium was again sterile filtered (0.22 μm) within the chamber. Anoxic phosphate-buffered saline (PBS) was generated by bubbling with nitrogen gas as above followed by overnight equilibration to the atmosphere in the anaerobic chamber.

To expose cells to anoxia, normoxic cell culture medium was removed in a biosafety cabinet, the 96-well plate was transferred into the anaerobic chamber through a pass box, and then the cells were washed three times with anoxic PBS. After the washes, cells were bathed in 200 μl per well of anoxic cell culture medium. Cells were maintained at 30°C using the built-in incubator in the anaerobic chamber. For recovery samples, cells were removed from the anaerobic chamber and washed three times with normoxic 1× PBS followed by incubation in normoxic complete cell medium in an incubator at 30°C under normal atmospheric conditions.

For some experiments, it was essential to have normoxic and anoxic samples in the same 96-well plate for imaging. In these circumstances, normoxic conditions were maintained in wells while the plates were within the anoxic chamber by sealing them with aluminum foil plate seals (FCS-25, AlumaSeal^®^ CS™, Excel Scientific, Victorville, CA, USA) under normoxic conditions with 100 μl of normoxic complete cell medium just prior to placing the plate into the anaerobic chamber. Sealed normoxic wells maintained aerobic conditions (80% saturation with O_2_ or greater) for up to 24 h when sealed in this manner and placed within the anaerobic chamber (data not shown).

### Immunofluorescence staining

Immunofluorescence staining and imaging were performed as recently described (Marmolejo et al., 2026). Briefly, cells were grown on 96-well optical plates (Cellvis, P96-1.5P) with a seeding density of 1.5×10^4^ cells/well. Cells were exposed to experimental conditions, including anoxia, 2 mM hydroxyurea (ab142613, Abcam, Waltham, MA, USA), 8 μM AZ20 (an inhibitor of ATR; HY-15557, MedChemExpress, Monmouth Junction, NJ, USA), or 0.01 μM AZD0156 (an inhibitor of ATM; HY-100016, MedChemExpress). If cells were exposed to inhibitors, they were given a pre-dose of inhibitors for 1 h prior to being moved to anoxic conditions and continually exposed to the drug during anoxia. During the final 15 min of exposure, cells were exposed to a pulse of 20 μM 5-ethynyl-2′-deoxyuridine (EdU; 900584, Sigma-Aldrich, St Louis, MO, USA). After exposure to EdU, cells were washed three times with 1× PBS and fixed with 4% paraformaldehyde (PFA) in PBS for 15 min. Fixed cells were stored at 4°C in PBS for up to 24 h before staining. Cells were permeabilized using ice-cold methanol for 10 min at 4°C and washed 3 times with 1× PBS at room temperature. Cells were incubated in 3% bovine serum albumin (BSA; A3294, Sigma-Aldrich) in 1× PBS for 10 min prior to initiation of the Click-iT reaction for EdU fluorophore conjugation using the Click-iT cell reaction buffer kit (C10269, Invitrogen, Carlsbad, CA, USA) and Alexa Fluor™ 647 azide (1 μg/ml; A10277, Invitrogen) according to the manufacturer's guidelines. Cells were incubated for 30 min at room temperature with constant rocking. After the Click-iT reaction, cells were washed with 3% BSA in PBS, followed by three washes in 1× PBS. Cells were blocked with 1% BSA in PBS for 1 h at room temperature on a bench top rocker. Following blocking, cells were incubated in rabbit polyclonal anti-zebrafish H2A.X phospho-Ser139 (1:500; 89424-450, VWR, Radnor, PA, USA) antibody overnight at 4°C with constant agitation. Following the overnight incubation with primary antibody, cells were washed three times in 1× PBS at room temperature and co-stained with 5 μg/ml DAPI (ab285390, Abcam) and an anti-rabbit-IgG Alexa Fluor 488-conjugated antibody in 1% BSA (1:1000; ab150077, Abcam) for 1 h at room temperature on a bench rocker. Finally, cells were washed three times with 1× PBS and imaged in 1× PBS.

### Quantitative image-based cytometry and analysis

Immuno-stained cells were imaged on a fully automated ImageXpress Micro Confocal Imaging System (Molecular Devices, San Jose, CA, USA) following methods previously described (Marmolejo et al., 2026). Widefield images were captured using a 20× Ph1S Plan Fluor ELWD ADM 0.45 NA objective and an sCMOS camera. Following image acquisition, CellProfiler (Broad Institute, Cambridge, MA, USA) was used for image analysis (Stirling et al., 2021). DAPI-stained images were used to create a nuclear mask for nuclear intensity measurements, and the mean intensity values for each marker were measured within the nuclear mask. DNA content was determined using DAPI integrated intensity.

### Live-cell imaging

Cells were grown on 96-well optical plates (Cellvis, P96-1.5P) with a seeding density of 1.5×10^4^ cells/well. Cells were then transferred to anoxic conditions as described above in 100 μl of anoxia medium. To maintain anoxic conditions during live imaging, two drops of anoxic mineral oil (Cumberland Swan, Smyrna, TN, USA) were floated on top of each well using a sterile disposable pipette before sealing with transparent sealing tape. This maintained anoxic conditions for at least 24 h while the plate was outside of the anaerobic chamber (data not shown). Brightfield images were taken every 15 min for 24 h on an Olympus IX83 inverted research microscope (Evident Scientific, State College, PA, USA). Plates were then removed from the microscope and returned to normoxic conditions by pipetting normoxic medium into the bottom of the well and washing out the mineral oil by overflowing the wells with normoxic media. This floated the anoxic mineral oil up and out of the wells and prevented exposing cells to oil which made imaging difficult. The plates were placed back onto the microscope and recovery from anoxia was documented with images taken every 15 min for 24 h. Final images were processed in ImageJ where the number of total cells and morphological dead cells were counted at 2-h timepoints (ImageJ 1.54p, National Institutes of Health, USA). Dividing cells and their daughter cells were tracked through exposure to anoxia and through reoxygenation and recovery.

### Statistical analysis

For all comparisons of proportional data, values were normalized and transformed through the arcsine square root transformation (*y*=arcsin(√*x*)) before one-way ANOVA with Dunnett's post hoc test was used to determine significance between means of individual treatments compared to normoxic controls.

## Supplementary Material


