# Supplementary material for: Anoxia tolerant DNA replication is supported by ATR kinase in the annual killifish Austrofundulus limnaeus
Source: J Cell Sci. 2026 Jul 27;139(14):jcs264892. doi: 10.1242/jcs.264892 (PMC13490736; doi:10.1242/jcs.264892)
Supplement: Supplementary file 1 [file joces-139-264892_review_history.pdf]

Journal of Cell Science

Anoxia tolerant DNA replication is supported by ATR kinase in the annual killifish *Austrofundulus limnaeus*

Riley Roth-Carter, Erin Helms, Joshua C. Saldivar and Jason Podrabsky

DOI: 10.1242/jcs.264892

Editor: Renata Basto

Review timeline

Original submission:	18 March 2026
Editorial decision:	10 June 2026
First revision received:	12 June 2026
Accepted:	17 June 2026

Original submission

First decision letter

MS ID#: jcs.264892

MS TITLE: Anoxia Tolerant DNA Replication is Supported by ATR Kinase in the Annual Killifish Austrofundulus limnaeus

AUTHORS: Riley Roth-Carter; Erin Helms; Joshua C Saldivar; Jason Podrabsky

ARTICLE TYPE: Research Article

Dear Dr Roth-Carter,

Let me start by apologising for the delayed decision. Unfortunately, reviewers are very busy and things took longer than expected.

We have now reached a decision on the above manuscript.

As you will see, the reviewers gave favourable reports but one reviewer raised very minor critical points that will require amendments to your manuscript. I hope that you will be able to carry these out because I would like to be able to accept your paper, depending on further comments from reviewers.

Reviewer 1

SUMMARY OF THE ADVANCE MADE IN THIS PAPER AND ITS POTENTIAL SIGNIFICANCE TO THE FIELD

In this manuscript the authors use flow cytometry to explore the impact of long-term anoxic incubation on cell cycle regulation in WS40NE immortalized cells from the Killifish. Using DNA levels they conclude that unlike anoxia sensitive cells these cells remain in the G1 phase for long periods after the first 24 hrs of anoxia and rapidly enter S phase after reoxygenation. Furthermore, they use ATR and ATM inhibitors to determine that the response is more dependent on ATR signalling. Î^{3}H2AX levels are used to assess DNA damage by its ability to bind to double stranded DNA. They report that increased Î^{3}H2AX levels are due to replication stress rather than DNA damage. This seems like a well conceived study but the n = 3 replicates seem rather low. Otherwise, an important contribution since the maintenance of G1 phase during anoxia is novel.

© 2026. Published by The Company of Biologists under the terms of the Creative Commons Attribution License (https://creativecommons.org/licenses/by/4.0/).

SUGGESTIONS TO AUTHORS

Major comments [Please request additional experiments only if they are essential for supporting the conclusions; authors should be encouraged to highlight any claims that are preliminary or speculative, or to discuss any pitfalls or alternative interpretations in a 'Limitations' section]

Minor comments. I only have minor issues.

Cell lines don't always behave to stressors as cells in in vivo. May be worth pointing this out just in case someone demonstrates in the future that this is the case for these cells.
Title: correct "the"
Page 2, line 60: change to "double stranded"
Figures 5 and 6 should be re-titled to indicate that these are the same enzymes and to better point-out that 5 is 24 hrs of normoxia and 6 is anoxic. As it is it looks as if Fig 5 has a normoxic exposure and then maybe an anoxic exposure, Fig 6 seems to have an anoxic exposure followed by a normoxic one.

Reviewer 2

SUMMARY OF THE ADVANCE MADE IN THIS PAPER AND ITS POTENTIAL SIGNIFICANCE TO THE FIELD

The paper investigates cell cycle and DNA damage response under anoxic conditions leveraging a unique cellular model derived from a non-canonical model organism. Annual killifish embryos are extremely resistant to anoxia. Here the authors show that their cells in vitro remain cycling for at least 48 hours despite accumulation of G-H2AX. Using small molecule inhibitors, the authors demonstrate a causal role of ATR in promoting genome integrity during anoxia.
I do not see any flaws in the paper.

SUGGESTIONS TO AUTHORS

Major comments [Please request additional experiments only if they are essential for supporting the conclusions; authors should be encouraged to highlight any claims that are preliminary or speculative, or to discuss any pitfalls or alternative interpretations in a 'Limitations' section]

Minor comments

First revision

Author response to reviewers' comments

We would like to thank the reviewers for taking the time to review this manuscript and for providing constructive suggestions for improvement. We have made the changes as suggested and hope that the manuscript is now suitable for publication. Our point-by-point response to the reviewers are listed below.

Comments from the Reviewers:

Reviewer 1: SUMMARY OF THE ADVANCE MADE IN THIS PAPER AND ITS POTENTIAL SIGNIFICANCE TO THE FIELD

In this manuscript the authors use flow cytometry to explore the impact of long-term anoxic incubation on cell cycle regulation in WS40NE immortalized cells from the Killifish. Using DNA levels they conclude that unlike anoxia sensitive cells these cells remain in the G1 phase for long periods after the first 24 hrs of anoxia and rapidly enter S phase after reoxygenation. Furthermore, they use ATR and ATM inhibitors to determine that the response is more

© 2026. Published by The Company of Biologists under the terms of the Creative Commons Attribution License (https://creativecommons.org/licenses/by/4.0/).

dependent on ATR signalling. γH2AX levels are used to assess DNA damage by its ability to bind to double stranded DNA. They report that increased γH2AX levels are due to replication stress rather than DNA damage. This seems like a well conceived study but the n = 3 replicates seem rather low. Otherwise, an important contribution since the maintenance of G1 phase during anoxia is novel.

We thank the reviewer for their favorable assessment of the study.

SUGGESTIONS TO AUTHORS

Major comments [Please request additional experiments only if they are essential for supporting the conclusions; authors should be encouraged to highlight any claims that are preliminary or speculative, or to discuss any pitfalls or alternative interpretations in a 'Limitations' section]

Minor comments. I only have minor issues.

Cell lines don't always behave to stressors as cells in in vivo. May be worth pointing this out just in case someone demonstrates in the future that this is the case for these cells.

We appreciate this view and agree it is an important reminder to readers. We have added an additional sentence to the Discussion (lines 250-252) stating that this stress response is cell line specific and continued work is needed to confirm in whole embryo model.

Title: correct "the"

“the” has been capitalized in title as suggested

Page 2, line 60: change to "double stranded"

Changed to "double stranded” as suggested

Figures 5 and 6 should be re-titled to indicate that these are the same enzymes and to better point-out that 5 is 24 hrs of normoxia and 6 is anoxic. As it is it looks as if Fig 5 has a normoxic exposure and then maybe an anoxic exposure, Fig 6 seems to have an anoxic exposure followed by a normoxic one.

Titles have been changed to better reflect that time courses of normoxia and anoxia in each figure along with indicating the same enzyme inhibition in both figures.

Reviewer 2: SUMMARY OF THE ADVANCE MADE IN THIS PAPER AND ITS POTENTIAL SIGNIFICANCE TO THE FIELD

The paper investigates cell cycle and DNA damage response under anoxic conditions leveraging a unique cellular model derived from a non-canonical model organism. Annual killifish embryos are extremely resistant to anoxia. Here the authors show that their cells in vitro remain cycling for at least 48 hours despite accumulation of G-H2AX. Using small molecule inhibitors, the authors demonstrate a causal role of ATR in promoting genome integrity during anoxia.

I do not see any flaws in the paper.

We wish to thank the reviewer for their kind assessment of this paper.

SUGGESTIONS TO AUTHORS

Major comments [Please request additional experiments only if they are essential for supporting the conclusions; authors should be encouraged to highlight any claims that are preliminary or speculative, or to discuss any pitfalls or alternative interpretations in a 'Limitations' section]

© 2026. Published by The Company of Biologists under the terms of the Creative Commons Attribution License (https://creativecommons.org/licenses/by/4.0/).

Minor comments

Second decision letter

MS ID#: jcs.264892R1

MS Title: Anoxia Tolerant DNA Replication is Supported by ATR Kinase in the Annual Killifish Austrofundulus limnaues

Authors: Riley Roth-Carter; Erin Helms; Joshua C Saldivar; Jason Podrabsky

Article Type: Research Article

Dear Dr Roth-Carter,

I am happy to tell you that your manuscript has been accepted for publication in Journal of Cell Science, pending standard publication integrity checks.

© 2026. Published by The Company of Biologists under the terms of the Creative Commons Attribution License (https://creativecommons.org/licenses/by/4.0/).
